# Observation of a half-illuminated mode in an open Penrose cavity

**DOI:** 10.1038/s41598-022-13963-y

**Published:** 2022-06-13

**Authors:** Juman Kim, Jinuk Kim, Jisung Seo, Kyu-Won Park, Songky Moon, Kyungwon An

**Affiliations:** 1 Department of Physics and Astronomy and Institute of Applied Physics, Seoul National University, Seoul, 08826 Korea; 2 Research Institute of Mathematics, Seoul National University, Seoul, 08826 Korea; 3 Faculty of Liberal Education, Seoul National University, Seoul, 08826 Korea

**Keywords:** Optical physics, Nonlinear phenomena

## Abstract

The illumination problem in mathematics questions the existence of a bounded region in which light rays from a point light source do not illuminate the whole region. Since Penrose disproved the illumination problem with elliptical reflective boundaries, the interest has mostly remained in ray optics mainly because there can be no completely dark region for light waves due to diffraction. Here, in a two-dimensional Penrose cavity with elliptical boundaries, we report experimental observation of a symmetry-broken mode in the long-wavelength regime with the half of the cavity region with reflection symmetry almost unilluminated in the steady state. The half-illuminated mode (HIM) was observed in an acoustic cavity by using the schlieren method. The HIM originated from the coherent superposition of near-degenerate modes, among which two scarred modes with opposite parities played a major role. The illuminated part of the HIM could be even flipped by choosing different coefficients in the coherent superposition of the participating modes. The HIM of the Penrose cavity provides new perspective to the illumination problem in an open system.

## Introduction

**Figure 1:**
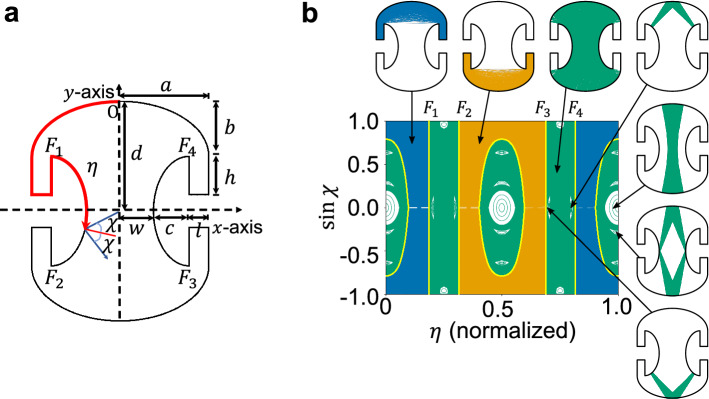
Geometry of the Penrose cavity and its phase space. (**a**) The shape of the Penrose cavity. Arc length $\eta$ is measured along the circumference from *O* counter-clockwise. The foci of top and bottom half-ellipses are denoted as ${F}_{1}$, ${F}_{2}$, ${F}_{3}$, and ${F}_{4}$, respectively. (**b**) There are three divided chaotic regions (colored in orange, green and blue, respectively) in the PSOS of the Penrose cavity. The solid yellow lines mark the boundaries between chaotic regions, and the positions of the foci are labeled on the PSOS. White regions represent island structures. From upper left to the lower right, chaotic orbit in the blue region, chaotic orbit in the orange region, chaotic orbit in the green region, V-shaped orbit in the upper half, axial orbit, diamond orbit and V-shaped orbit in the lower half are shown with arrows indicating the corresponding points on the PSOS.

A Penrose-unilluminable-room cavity is an optical resonator with the boundary shape suggested by Penrose in response to the illumination problem^1^ put forth by Ernst Straus. The illumination problem questions whether it is possible to illuminate every region with a point light source from any (or at least one) point in the region surrounded by mirrored walls^2,3^. The Penrose-unilluminable-room cavity, or the Penrose cavity in short from now on, has elliptical top and bottom boundaries and elliptical-mushroom-shaped protruded boundaries in the middle with *xy*-reflection symmetry, as shown in Fig. 1a, allowing unilluminated points which cannot be reached by classical ray trajectories emitted from some points in the cavity^4^. Since the Penrose’s suggestion, its various extensions disproved the illumination problem—answering ‘No’ to the question—for smooth^5^ as well as polygonal boundaries^6–9^.

The illumination problem belongs to a broader class of problems dealing with billiard systems with divided phase space^10^. In particular, the Penrose cavity has multiple ergodic regions divided by separatrices in its phase space. The classical rays launched from one region cannot be transported to the other regions due to the separatrices. For wave optics, on the other hand, it is mathematically forbidden to have completely unilluminated points in any bounded region^11^. The light wave easily gets around an obstacle by diffraction and reaches the forbidden regions of the geometrical optics. The evolution of a Gaussian light wave emitted from a point source inside the Penrose cavity as a closed system was demonstrated with finite difference time domain (FDTD) method, resulting in the whole region to be illuminated with *xy*-reflection symmetry in the end^12^. Moreover, due to the diffraction, chaotic resonator modes are not localized within the divided regions, but spread across the separatrices^13^.

One may note that the illumination problem, whether in terms of ray or wave optics, has mostly been addressed for closed cavities with perfectly reflecting walls. If one considers any physical realization of the Penrose cavity, however, one has to take the cavity decay into account. The system is then an open system described by non-Hermitian Hamiltonian with eigenvalues $E_n/\hbar =\omega _n -{\mathrm {i}}\gamma _n$, where $\hbar$ is the reduced Planck constant and the cavity resonances $\omega _n$ have linewidths $2\gamma _n$. As a natural extension of the illumination problem to open systems, it is reasonable to ask whether the resonance modes are still distributed in the Penrose cavity with *xy*-reflection symmetry as in the case of the closed cavity.

In this paper, we address this problem by performing actual experiments on an acoustic Penrose cavity as an analogue of a wave optical system, measuring the intensity distributions of resonant acoustic modes. We observed almost all modes are distributed in the cavity keeping the *xy* reflection symmetry due to the aforementioned wave nature and we could associate them with corresponding ray dynamics. However, we have also observed a half-illuminated mode (HIM) with broken reflection symmetry, filling only a half of the symmetric cavity region, leaving the rest half almost unilluminated in the steady state. We could even flip the illuminated region over by adjusting the position of the external driving field relative to the cavity.

Existence of such HIM’s with broken reflection symmetry has not been expected nor reported. We explain the observed HIM in terms of coherent superposition of multiple near-degenerate modes with overlapping linewidths around the external driving frequency. Such composite-mode formation is only possible in open systems. We also show that scarred modes with opposite parities play a major role in the formation of the HIM.

It is noteworthy that an arbitrary spatial pattern can be created at any instant by coherent superposition of eigenstates in a closed cavity owing to the completeness of the eigenstates. However, such a pattern cannot be maintained in time because these eigenstates oscillate at different frequencies. The HIM’s that we observed is a steady-state mode composed by near-degenerate opposite-parity modes in an open Penrose cavity, which is also a non-integrable system. Analytic formulae or algorithms are thus not available, making it almost impossible to predict its existence a priori, contributing to the reason why HIM’s have not been predicted before.

## Results

The acoustic Penrose cavity we consider has the following geometry. Its top and bottom curves are ellipses, whose length of semi-major axis and semi-minor axis is *a* and *b*, respectively, and the length and width of four arms are *h* and *l*, respectively, as shown in Fig. 1a. The points $F_{n}(n=1,2,3,4)$ represent foci (or values of $\eta$ there) of half ellipses whose focal length is $f=\sqrt{a^2-b^2}$. The length of the semi-minor axis of the concave side ellipses and the vertical half height of the cavity are represented with *c* and *d*, respectively. The length 2*w* is the distance between the side ellipses. The cavity boundary has mirror-symmetry along the *x* and *y* axes. Parameter values are $(a,b,c,w,d,h,l,f)=(4.80,3.00,1.95,1.80,6.00,2.10,1.05,3.75)$ mm in the experiment as well as in the ray simulation. An aluminum cavity is precision machined according to this specification with tolerance of 10 $\mu$m (corresponding to $\lambda /100$ with $\lambda$ the acoustic wavelength). The total arclength *L* of the cavity boundary is $(61.56\pm 0.01)$ mm.

The characteristic ray dynamics is easily identified in the Poincaré surface of section (PSOS)^14^. Figure 1b shows the PSOS of the billiard of Penrose cavity (a closed system)^15^, plotted by employing the Birkhoff coordinates $(\eta , \chi )$, where $\eta$ is the arclength measured from *O* counter-clockwise in Fig. 1a and $\chi$ is the incident angle at the bouncing point of randomly launched particles. The blue(orange) region in the PSOS corresponds to the chaotic trajectories with the arclength $\eta$ confined to $0<\eta <{F}_{1}$, ${F}_{4}<\eta < 1$ ($F_{2}<\eta <F_{3}$) and the green region corresponds to those confined to ${F}_{1}<\eta <{F}_{2}$ and ${F}_{3}<\eta <{F}_{4}$. The white regions in the green chaotic sea are regular regions or islands, corresponding to stable periodic orbits such as axial, diamond-shaped, and V-shaped orbits^15^. The chaotic regions highlighted in different colors are ray-dynamically separated by dynamical barriers (yellow solid lines), which originate from the properties of the ellipse, and therefore, there is no classical flow transported across the barriers.

### Observation of resonant mode patterns

**Figure 2:**
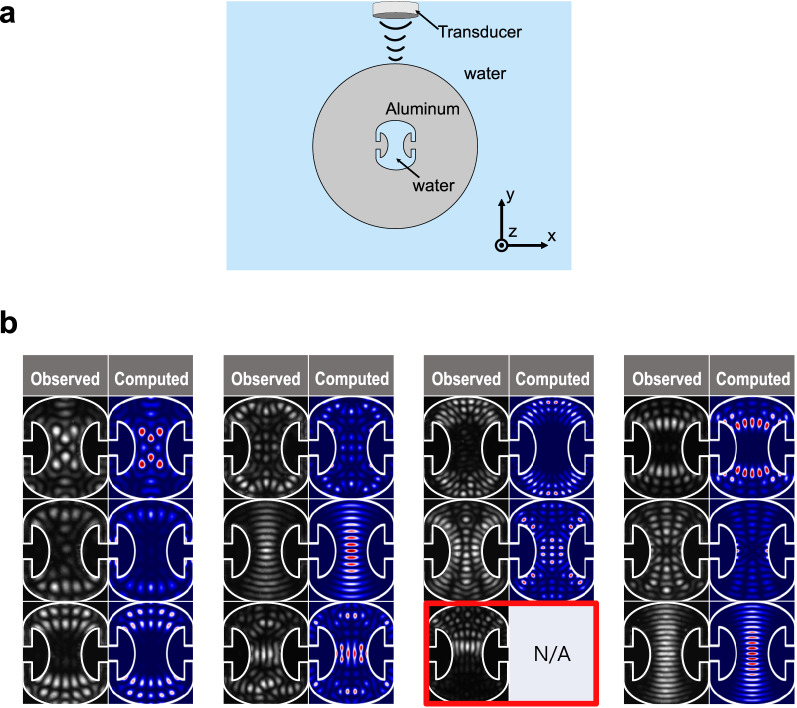
Comparison of observed mode patterns with computed ones. (**a**) Mode excitation arrangement. The gray shaded region is aluminum and light blue shaded regions correspond to water. (**b**) Images of the experimentally observed acoustic resonant modes compared with numerically computed ones. The resonant frequencies range from (771.3 ± 0.1) kHz to (1262.3 ± 0.3) kHz and their Q factors vary from 290 ± 30 to 1000 ± 100. Among the observed modes, an HIM is highlighted with red box.

To investigate the resonant modes of the Penrose cavity, we fabricated an acoustic Penrose cavity in the center of an aluminum cylinder along its axis. The cylinder, supported with a thin thread horizontally, is immersed in a water tank to fill the cavity with water. The cross sectional view of the cavity and the location of an acoustic transducer is illustrated in Fig. 2a. The outer diameter of the circular cylinder is 40 mm with tolerance of 10 $\upmu$m and the length of the cylinder is 80 mm, which is sufficiently larger (about seven times) than the length over which acoustic modes are excited in the cavity, making sure that we are dealing with a two dimensional system. The ultrasound in the range from 700 to 1300 kHz is generated with an immersion ultrasonic transducer (ISL-0504-GP, Technisonic) placed above the cylinder and directed vertically downward ($-y$ direction) in order to drive the acoustic resonant modes inside the cavity. This frequency range corresponds to the size parameters $L/\lambda =29{-}54$, where *L* is the arclength of the cavity and $\lambda$ is wavelength of the acoustic wave. In this long-wavelength regime, the wavelike behaviour such as diffraction is dominant and the resonant modes are expected to be distributed more or less over the entire region symmetrically. The position of the transducer is controlled with a motorized linear stage in the horizontal direction (*x* direction). The vertical distance from the cylinder to the transducer is fixed at (32.1 ± 0.3) mm.

The resonant modes are imaged using the schlieren imaging method^16,17^ with a 532 nm cw laser. Similar techniques have been used to observe the mode patterns in integrable systems^18^, non-integrable systems^19^ and near-exceptional-point systems^20^ in a non-perturbing manner. The detail of our schlieren imaging setup is explained in Methods. The observed resonant modes and computed eigenmodes corresponding to them are shown in Fig. 2b side by side. The observed modes can be well matched with the eigenmodes computed by using a finite-element-simulation code (COMSOL Multiphysics) except the one highlighted with red box. This unusual mode pattern with broken reflection symmetry cannot be matched with any of the computed eigenmodes.

### A-half-illuminated modes

In an open system, multiple nondegenerate resonance modes can be excited simultaneously by an external driving field of frequency $\omega _d$ when their resonance frequencies $\omega _n$ are close to the driving frequency compared to their linewidths $2\gamma _n$. As a result, a composite mode $\Psi$, a linear superposition of these eigenmodes with complex coefficients $c_n$, can be excited in the steady state. In our case, the composite mode $\Psi ({\mathbf {r}},t)$ is expressed as,

1 $$\begin{aligned} \begin{array}{c} \Psi ({\mathbf {r}},t)=\psi ({\mathbf {r}}){\mathrm {e}}^{{\mathrm {i}}\omega _{d}t}, ~\psi ({\mathbf {r}})\simeq \psi _{N}({\mathbf {r}})=\sum _{n=1}^{N}c_{n}\phi _{n}({\mathbf {r}}) \end{array} \end{aligned}$$

where $\phi _{n}({\mathbf {r}})$ are complex acoustic quasi-eigenstates of the wave equation described in Methods, $\psi ({\mathbf {r}})$ is complex wavefunction of the composite mode and $\psi _{N}$ is a truncated superposition of *N* quasi-eigenmodes ($N=20$ is used for the HIM below). The intensity $I({\mathbf {r}})$ of the resulting composite mode is given by $I({\mathbf {r}})\propto \left| \psi ({\mathbf {r}})\right| ^{2}$.

**Figure 3:**
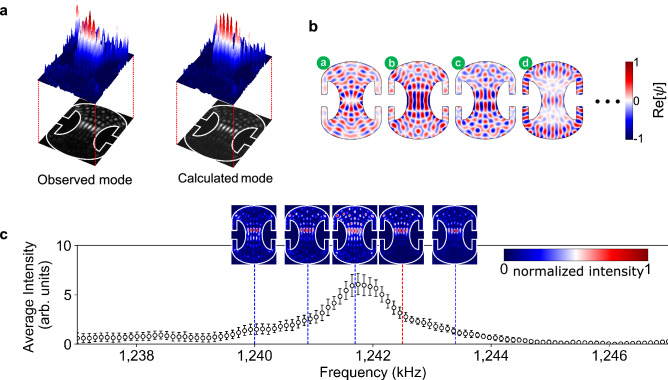
Numerical reproduction and the excitation spectrum of HIM. (**a**) The image of the HIM and the numerical reproduced one from superposition of near-degenerate resonant modes. The white solid lines represent the boundary between aluminum and water. Animation is also available (Supplementary Movie 1). (**b**) The mode patterns of the four near-degenerate resonant modes most contributing to HIM. The real parts of the field distributions of complex eigenstates in the fluid region inside the Penrose cavity are shown. (**c**) The excitation spectrum of the HIM (black open circles). The error bar indicates one-standard deviation at each sample mean. Mode patterns at five representative frequencies are also shown. The red dotted vertical line indicates the frequency at which the HIM is observed.

In numerical simulations, the complex coefficients $c_{n}$ of the overlapping resonant modes are optimized to reproduce the observed intensity distribution (Fig. 3a). The HIM image is processed by subtracting a background (obtained with the transducer off) from the raw image (with the transducer on)^21^. We optimize the complex coefficients by using the differential evolution algorithm^22^ to maximize the overlap integral ${\mathscr {O}}$ between square root of the observed intensity distribution $\sqrt{I({\mathbf {r}})}$ and the computed $|\psi _{N}({\mathbf {r}})|$ of Eq. (1), with ${\mathscr {O}}$ defined as

2 $$\begin{aligned} {\mathscr {O}}(\{ c_n \}) = \frac{(\iint _S \sqrt{I(x, y)}|\psi _{N}(x, y; c_{n})| \,dxdy)^{2}}{\iint _S I(x,y) \,dxdy \iint _S |\psi _{N}(x, y; c_{n})|^{2} \,dxdy} \end{aligned}$$

where the subscript *S* indicates integration over the Penrose cavity region (water filled) only. Because of the normalization, we have $0\le {\mathscr {O}}\le 1$. The ${\mathscr {O}}$ value obtained for the observed and computed ones in Fig. 3a is 0.76.

The main reason for the modest value of the overlap integral is the imperfect reproduction of the quasi-eigenstates in the simulation. The overlap integral between an observed quasi-eigenstate and the computed one corresponding to it ranges from 0.7 to 0.8. The discrepancy is mostly due to the diffraction of the optical beam near the cavity boundary in schlieren imaging and the nonlinearity between the acoustic pressure field and the refractive index change for high pressure fields. As a result, the overlap integral in Fig. 3 is also limited to a similar level of 0.76.

**Figure 4:**
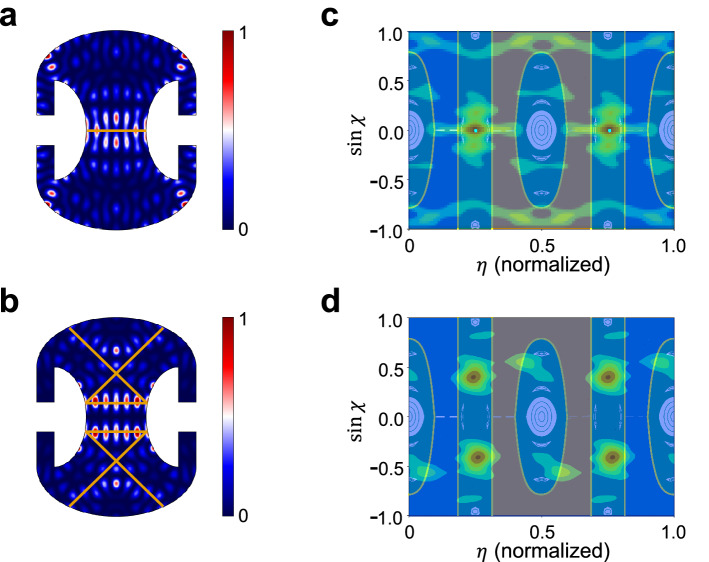
Scarred and quasi-scarred modes contributing to the HIM. (**a**) The intensity distribution of mode b (a scarred mode) and (**b**) that of mode c (a quasi-scarred mode), as labeled in Fig. 3b, and their corresponding ray trajectories (orange solid lines), respectively. (**c**) Husimi distribution of mode b overlapped on the PSOS. (**d**) The same for mode c. The scarred mode in (**a**) can be associated with an unstable periodic orbit [marked by cyan-colored points in (**c**)]. The quasi-scarred mode in (**b**) has no corresponding periodic orbit.

From the result of coefficient optimization for the largest overlap integral, we interpret $|c_{n}|^{2}$ divided by sum of $|c_{n}|^{2}$ from *n*=1 to *n*=20 as contribution of *n*th mode. Fig. 3b shows the real part of the field (not intensity) distributions of the four most-contributing resonant modes (labeled a, b, c and d) whose lineshapes are overlapping each other. For numerical simulations, we used the actual boundary profile of the Penrose cavity in order to enhance the coefficient optimization because the calculated modes are sensitive to geometrical defects, such as rounding of corners. The boundary profile was obtained from the actual cross sectional image of the cavity. Modes a, b and c (mode d) correspond to the ray trajectories trapped inside green regions (orange and blue regions) in Fig. 1b. Modes b and c (whose contributions are 12.8% and 12.5% respectively), significantly contributing to the HIM, are a scarred mode^23,24^ and a quasi-scarred mode^25,26^, respectively, where the former has even parity and the latter has odd parity along the *y*(vertical) axis. Their mode distributions (calculated for the designed shape, not the actual shape), corresponding classical trajectories and Husimi distributions are shown in Fig. 4. The scarred mode is strongly localized on an unstable periodic orbit whereas the quasi-scarred mode has no underlying periodic orbit. These (quasi-)scarred modes do not occur in the closed cavity obviously, supporting that the HIM cannot occur in a closed system.

**Figure 5:**
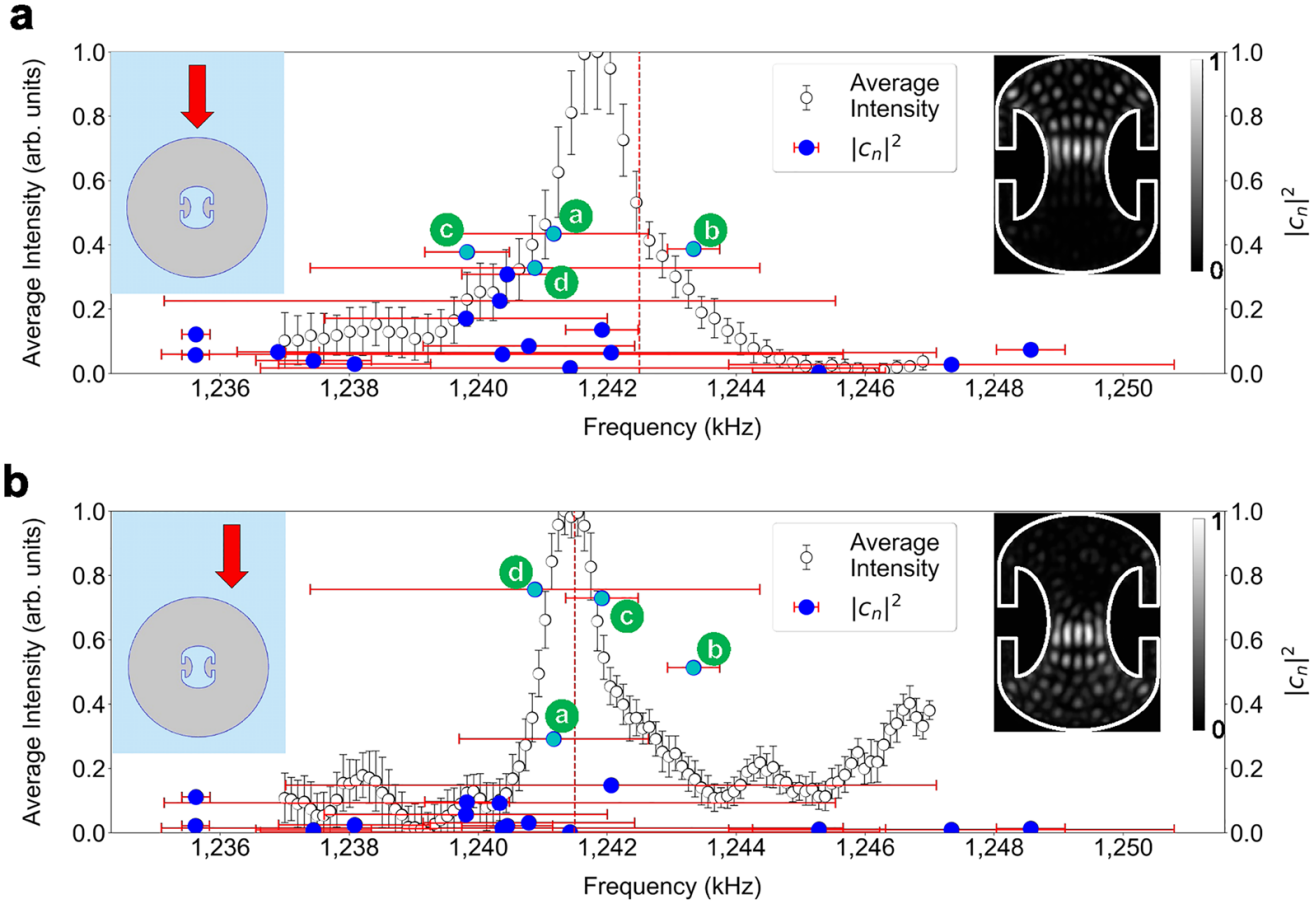
Flipping the half-illuminated region. The normalized spectrum of (**a**) the HIM and (**b**) the inverted HIM. The left and right insets are the excitation position and the steady-state mode pattern, respectively in each case. The peak frequencies are (1241.713 ± 0.033) kHz for (**a**) and (1241.410 ± 0.056) kHz for (**b**), respectively. The HIM (inverted HIM) is excited at 1242.5 kHz (1241.5 kHz). The excitation frequencies are indicated as red dashed lines. The blue dots represent the eigenmodes contributing to the HIM (inverted HIM) with the horizontal positions indicating their resonance frequencies and red horizontal bars showing their linewidths. Their vertical positions of the dots indicate the magnitude of corresponding $|c_n|^2$. The most contributing four modes, also shown in Fig. 3b as labelled a, b, c and d, are colored in cyan. The vertical error bar indicates one-standard deviation at each sample mean.

In Fig. 3c, the spectrum is obtained by summing up the intensity distribution of observed modes at each driving frequency and by plotting the result while scanning the driving frequency. The spectrum exhibits an asymmetric Fano-resonance-like profile^27^ (more clearly seen in Fig. 5) due to the coherent excitation of several resonant modes of low and high quality factors. The insets show the observed distributions at some of the driving frequencies (indicated by blue dashed lines) and the red dashed line indicates the driving frequency where the most optimal HIM image is obtained.

## Discussion

The magnitude and phase of the complex coefficients $\{c_n\}$ depend on the position of the source of the driving field. A different set of complex coefficients must result in a different composite-mode pattern. In Fig. 5, the spectra are measured at different excitation positions, in the center for **a** and about 8 mm translated horizontally from the center for **b**. The overall intensity or the integral of the raw spectrum in **b** is about 20% of that of **a**. The spectra in Fig. 5 are normalized for maximum to be 1 in order to clearly compare their line shapes. The vertical red dashed lines on the spectra in Fig. 5a,b indicate the driving frequencies where the composite modes in the right insets are observed. It is shown that the HIM can be inverted by displacing the transducer by (7.9 ± 0.4) mm (which is $40\%$ of aluminum shell radius) with the driving field directed vertically downward. The different spectra also show that the two HIM modes have different contributions from the overlapping quasi-eigenmodes at two excitation positions. In Fig. 5b, modes a, b, c and d are also found to be major components in the reverse order. Mode c indicates two modes with slightly different eigenfrequencies with almost the same mode pattern in Fig. 5a,b. The HIM and the inverted HIM occur at slightly higher frequencies than the peak frequencies for both excitation conditions.

In the HIM and the inverted HIM, the reflection symmetry with respect to *x* axis is broken. We have not observed any HIM’s with broken reflection symmetry with respect to *y* axis. It appears to be related to the fact that there exist no scar (or quasi-scar) modes distributed along the *y* direction. This observation clearly supports the essential role of scar modes in the formation of HIM’s.

Although the occurrence of HIMs is not a direct consequence of the dynamical barriers in the Penrose cavity, the dynamical barriers play an indirect role in that most of the participating modes including the four modes shown in Fig. 3 are localized to distinct regions separated by the dynamical barriers.

In a closed cavity, one can compose a highly asymmetric spatial pattern like an HIM at any instant by superposing eigenmodes since they form a complete set. However, these modes oscillate in time with different eigenfrequencies, and therefore at later time the asymmetric pattern cannot be maintained. The HIM that we have observed is a steady-state mode, not changing in time. The HIM requires the lineshapes of the participating quasi-eigenmodes, particularly two opposite-parity (quasi-)scar modes, to be overlapping with each other within their linewidths, which means the system must be open as well as non-integrable. Because of the non-integrability, no analytic formulae are available and thus it is almost impossible to predict existence of HIM in advance. This may explain why HIM’s have not been predicted nor observed before.

## Conclusion

We have investigated the resonant mode patterns in an acoustic Penrose cavity experimentally in the long-wavelength regime. Most of the observed resonant modes could be one-to-one matched with numerically computed ones for the cavity geometry while distributed in the cavity with *xy*-reflection symmetry as expected from the strong wave nature. However, we observed an unexpected half-illuminated mode with a broken-reflection-symmetry pattern, i.e., with the half of the cavity region unilluminated even in the long-wavelength regime. The observed HIM could be explained by coherent superposition of multiple near-degenerate modes, including two scarred modes with different parities, simultaneously excited by an external driving field. The observed HIM could also be flipped by choosing different excitation position on the cavity and thus different coefficients in the superposition. Our results may find applications in on-off control of room acoustics or Wi-Fi distribution and can be used to extend the illumination problem to other types of open systems.

## Methods

### Obtaining eigenmodes and eigenvalues numerically

Acoustic resonant modes are obtained by solving the Helmholtz equation given by

3 $$\begin{aligned} (\nabla ^{2} + k_{\mathrm{{f}}}^{2})\psi =0 \end{aligned}$$

in the fluid region and the Cauchy–Navier equation given by

4 $$\begin{aligned} -\rho _{\mathrm{{s}}}\omega ^{2}{\mathbf {u}}= (\lambda + 2\mu )\nabla (\nabla \cdot {\mathbf {u}}) - \mu \nabla \times (\nabla \times {\mathbf {u}}), \end{aligned}$$

5 $$\begin{aligned} {\hat{n}}\cdot \nabla \psi= (-\rho _{\mathrm{{f}}}\omega ^{2}){\hat{n}}\cdot {\mathbf {u}}, \end{aligned}$$

in the solid region, where $k_{\mathrm{{f}}}=\omega /v_{\mathrm{{f}}}$ is the wavenumber of the fluid, $\psi$ the pressure in the fluid region, $v_{\mathrm{{f}}}$ the speed of sound in the fluid, $\lambda$ and $\mu$, Lamé’s first and second parameters, respectively, ${\mathbf {u}}$ the displacement vector in the solid, $\omega$ the angular frequency, $\rho _{\mathrm{{s}}}$ the density of the solid and $\rho _{\mathrm{{f}}}$ the density of the fluid. The waves in the inner fluid region, the solid region and the outer fluid region extending to infinity are coupled to each other through Eq. (5) at the boundaries. We employed the finite-element method for solving the wave equations with the following boundary conditions.

6 $$\begin{aligned}\sigma _{nn} = -\psi , \end{aligned}$$

7 $$\begin{aligned}\sigma _{nt} = 0. \end{aligned}$$

where *n* and *t* represent the boundary normal and tangential components of the stress tensor $\sigma$ defined as

8 $$\begin{aligned} \sigma _{ij} = \lambda (\nabla \cdot {\mathbf {u}})\delta _{ij} + \mu [(\nabla {\mathbf {u}})_{ij}+(\nabla {\mathbf {u}})_{ji}]\,(i,j=n,t) \end{aligned}$$

Equation (5) comes from the continuity of the displacement, Eq. (6) is the equilibrium condition between the stress and the pressure and Eq. (7) states there is no shear force in the water.

**Figure 6:**
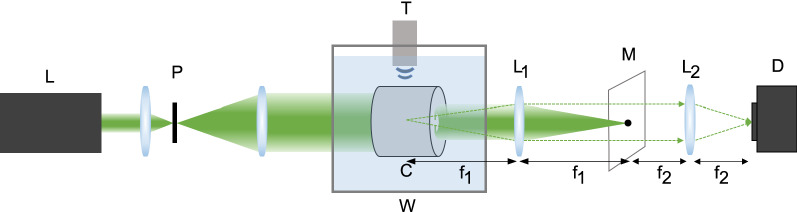
Schematic diagram of the schlieren imaging setup. L: 532 nm laser, P: pinhole, T: transducer, C: Penrose cavity, W: water tank, M: mask, D: detector. A 2*f*–$2f'$ imaging system (lenses L$_1$ and L$_2$ with $f_1=200$ cm and $f_2=75$ cm, respectively) is used to image the intensity patterns of acoustic resonant modes.

### Experimental setup

The schematic of the schlieren imaging setup is shown in Fig. 6. The 532 nm cw laser beam propagates through the water-filled cavity along its axis after expanded to cover the whole cavity region. The ultrasonic field generated by a transducer (ISL-0504-GP, Technisonic) is used to excite the resonant acoustic modes of the cavity. The acoustic modes introduce refractive index modulation in the water inside the cavity. We employ a 2*f*–$2f'$ imaging system to image the deflected laser light by the refractive index modulation. The nondeflected light is eliminated by a black dot in the mask located at the focal point of L$_1$ ($f_1=200$ cm). The deflected light containing the information of the acoustic modes emerges from the focal point of L$_1$ on the left, relayed to L$_2$ and then imaged on a charge-coupled device (acA4112-30um, Basler) located at the focal point of L$_2$ ($f_2=75$ cm). For the pressure of the acoustic wave achievable in our experiment, the light intensity imaged on the detector is proportional to the square of the pressure field associated with the cavity mode^28^. A sound absorber (Thinsulate, 3M) is laid on the bottom of the water tank to prevent unwanted reflection of acoustic waves. The ambient temperature is set at (19.3 ± 0.1) °C and the laser power fluctuation is stabilized with a proportional-integral-differential feedback loop.

## Supplementary Information

Supplementary Movie 1.

## Data Availability

The datasets generated during the current study are available from the corresponding author on reasonable request.
